# Spectral Analysis and Crystal Structures of 4-(4-Methylphenyl)-6-Phenyl-2,3,3a, 4-Tetrahydro-1H-Pyrido[3,2,1-jk]Carbazole and 4-(4-Methoxyphenyl)-6-Phenyl-2,3,3a, 4-Tetrahydro-1H-Pyrido[3,2,1-jk]Carbazole

**DOI:** 10.5402/2011/541082

**Published:** 2011-04-17

**Authors:** J. Kalyana Sundar, S. Natarajan, S. Chitra, Nidhin Paul, P. Manisankar, S. Muthusubramanian, J. Suresh

**Affiliations:** ^1^School of Physics, Madurai Kamaraj University, Madurai 625 021, India; ^2^Department of Industrial Chemistry, School of Chemistry, Alagappa University, Karaikudi 630 003, India; ^3^Department of Organic Chemistry, School of Chemistry, Madurai Kamaraj University, Madurai 625 021, India; ^4^Department of Physics, The Madura College, Madurai 625 011, India

## Abstract

The crystal structures of 4-(4-methylphenyl)-6-phenyl-2,3,3a,4-tetrahydro-1H-pyrido[3,2,1-jk]carbazole (**IIa**) and 4-(4-methoxyphenyl)-6-phenyl-2,3,3a,4-tetrahydro-1H-pyrido[3,2,1-jk]carbazole (**IIb**) were elucidated by single crystal X-ray diffraction. Compound (**IIa**), C_28_H_25_N, crystallizes in the triclinic system, space group P-1, with *a* = 8.936(2) Å, *b* = 10.490(1) Å, *c* = 11.801(1) Å, *α* = 102.69(5)^*°*^, * β* = 103.27(3)^*°*^, *γ* = 93.80(1)^*°*^, and *Z* = 2. The compound (**IIb**), C_28_H_25_NO, crystallizes in the monoclinic system, space group P2_1_/a, with *a* = 11.376(5) Å, *b* = 14.139(3) Å, *c* = 13.237(4) Å, *β* = 97.41(3)^*°*^, and *Z* = 4. In both the structures, the pyrido ring adopts a twist boat conformation and the carbazole molecule has the twisted envelope structure with C3 and C13 at the flap. No classical hydrogen bonds are observed in the crystal structures. Details of the preparation, structures, and spectroscopic properties of the new compounds are discussed.

## 1. Introduction

Many natural products with carbazole moiety are found to have antitumor properties [1]. The Schiff bases derived from 9-aminocarbazole showed notable activity as agrochemical fungicides [2]. Indolo [2,3-a] carbazole-based inhibitors were synthesized, and they displayed moderate inhibitory activities toward *Bacillus anthracis* and *Mycobacterium tuberculosis*, indicating that indolo [2,3-a] carbazoles could serve as promising leads in the development of new drugs to combat anthrax and tuberculosis infections [3]. Natural and synthetic carbazoles, either in a pure substituted or in an annellated substituted form, represent an important and heterogeneous class of anticancer agents. Many carbazole derivatives were tested for cytotoxic activity; some of them have entered clinical trials [4]. A series of N-alkylated 3,6-dihalogenocarbazoles exhibit fungicidal activity against *C. albicans* and the emerging pathogen *Candida glabrata* [5]. Although the carbazole motif has been characterized structurally over 700 times, tetrahydrocarbazoles are much rarer, and of the tetracyclic nature, including the pyrido ring, there are actually only six, with the closest being 5,6-Dihydro-8-methyl-4H-pyrazino (3,2,1-jk) carbazole [6]. This paper reports the preparation of two carbazole derivatives and their structural investigations.

## 2. Experimental

For the preparation of the compound (**IIa**), a mixture of 2-(3-oxo-1-(4-methylphenyl)-3-phenylpropyl)cyclohexanone (3 mmol) and phenylhydrazine hydrochloride (6 mmol) in ethanol (20 mL) was stirred for 2 hrs. After the completion of the reaction, the mixture was poured into excess water and the precipitate was filtered off and recrystallized from ethanol (Yield 0.71 g, 72%; m.p. 454 K). For the preparation of compound (**IIb**), a mixture of 2-(1-(4-methoxyphenyl)-3-oxo-3-phenylpropyl) cyclohexanone (3 mmol) and phenylhydrazine hydrochloride (6 mmol) in ethanol (20 mL) was stirred for 2 hrs. After the completion of the reaction, the mixture was poured into excess water and the precipitate was filtered off and recrystallized from ethanol [7] (Yield 0.78 g, 66%; m.p. 475 K).

### 2.1. Structure Determination and Refinement

**Figure 1:**
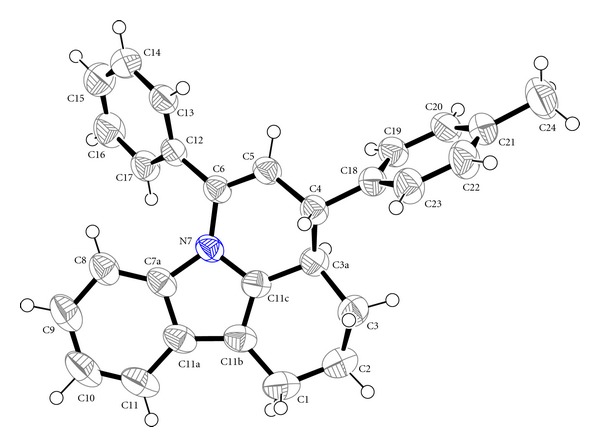
The molecular structure of compound (**IIa**) showing the atom numbering scheme. Displacement ellipsoids are drawn at 50% probability level, using ORTEP 3. Hydrogen atoms are drawn as spheres of arbitrary size.

**Figure 2:**
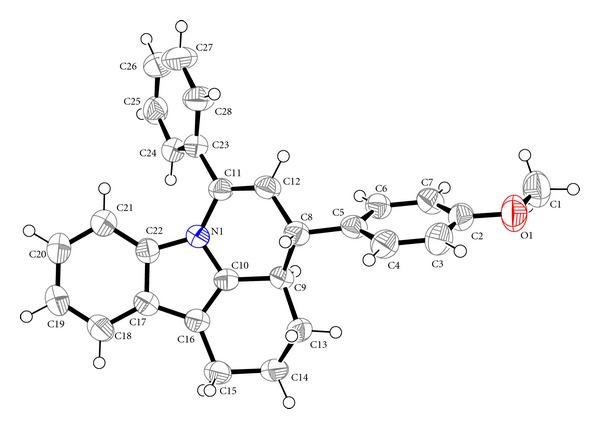
The molecular structure of compound (**IIb**) showing the atom numbering scheme. Displacement ellipsoids are drawn at 50% probability level, using ORTEP 3. Hydrogen atoms are drawn as spheres of arbitrary size.

Single crystal X-ray intensity data for compound (**IIa**) and (**IIb**) were collected using a Nonius CAD-4 MACH 3 diffractometer with MoK*α* (0.71073 Å) radiation at room temperature (293 K). The data reduction was performed with XCAD4 [2, 8]. An absorption correction was made using the *ψ*-scan method [9]. The structures of both compounds were solved by direct methods using SHELXS97 [10], and all the nonhydrogen atoms were refined anisotropically by full-matrix least-squares on *F* ^2^ taking all the unique reflections using SHELXL97 [11]. The hydrogen atoms were placed in their calculated positions and included in the refinement using the riding model with C–H = 0.93 Å (–CH) or 0.97 Å (–CH_2_) Å or 0.96 Å (–CH_3_) Å with *U*_iso_(H) = 1.2*U*_eq_ (parent C atom). The crystal data, experimental conditions, and structure refinement parameters for the compounds (**IIa**) and (**IIb**) are presented in Tables 1 and 2. Some important bond lengths and bond angles are given in Tables 3 and 4. The molecular structures of compound (**IIa**) and (**IIb**) showing the atom numbering scheme using ORTEP 3 [12] are given in Figures 1 and 2, respectively.

## 3. Results and Discussion

### 3.1. Synthesis of the Compounds

**Figure 3:**
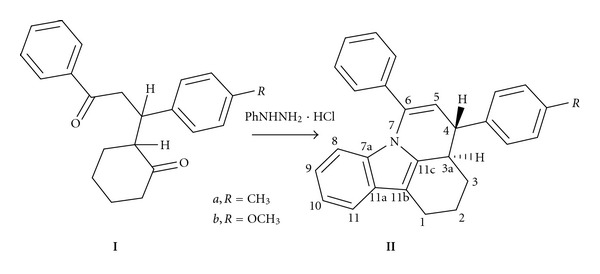
Preparation of **II**.

The title compounds, 4-(4-methylphenyl/4-methoxyphenyl)-6-phenyl-2,3,3a,4-tetrahydro-*1H*-pyrido[3,2,1-*jk*]carbazoles, have been prepared from the diketones, 2-(3-oxo-1-(4-methylphenyl/methoxyphenyl)-3-phenylpropyl) cyclohexanones following the procedure of Moskovkina and Thilichenko [7]. The scheme of the synthesis is shown in Figure 3. The reaction has yielded only one of the diastereomeric products, though two isomers are expected based on the fact that there are two stereogenic centers. Hence the reaction is diastereoselective giving the product with an equatorial arrangement for the aryl ring. The formation of the other diastereomer is difficult probably due to conformational and steric reasons. It can be noted that the reaction involves a domino transformation comprising a Fischer indole synthesis (which is a domino process in itself) and an intramolecular enamine formation.

### 3.2. Spectral Data

**Figure 4:**
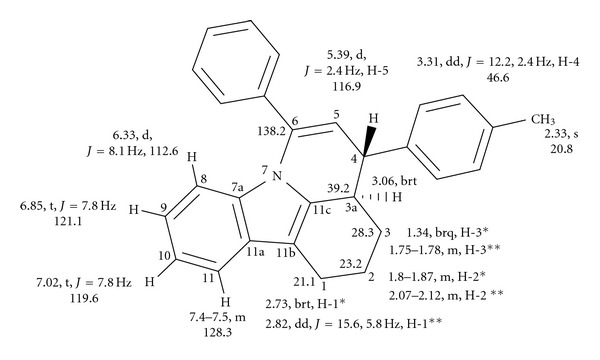
The assignment of different carbons and hydrogens for compound (**IIa**) based on the connectivity studies.

In the IR spectrum of (**IIa)**, bands appear at 3051 cm^−1^ (aromatic C–H stretching), 1567 cm^−1^ (olefinic C=C stretching), 2844 cm^−1^, and 2923 cm^−1^ (aliphatic C–H stretching). The identity of the above compound has been ascertained by NMR spectral data. A detailed analysis of the two-dimensional NMR spectra has helped to assign the hydrogens and carbons of interest in these compounds. The numbering for carbons and hydrogens given here for NMR data and crystal structure is for convenience and not exactly the correct way of numbering according to IUPAC rules. Compound (**IIa)** has a broad quartet at 1.34 ppm (1H, H-3_axial like_), a multiplet between 1.75–1.78 ppm (1H, H-3_equatorial like_), a multiplet between 1.80–1.87 ppm (1H, H-2_axial like_), a multiplet at 2.07–2.12 (1H, H-2_equatorial like_), a strong triplet with further fine coupling at 2.73 ppm (1H, H-1_axial like_), and a doublet of doublet at 2.82 ppm (1H, *J* = 15.6 & 5.8 Hz, H-1_equatorial_). The H-3a proton appears as a broadened triplet at 3.06 ppm with coupling constant *J* = 11.7 Hz, while H-4 proton appears as a doublet of doublet at 3.31 ppm (*J* = 12.2 & 2.4 Hz). A doublet appears at 5.39 ppm, (*J* = 2.4 Hz), which is assigned to H-5 proton. The H-8 proton appears as a doublet at 6.33 ppm (*J* = 8.1 Hz) followed by two triplets centering at 6.85 ppm and 7.02 ppm (*J* = 7.8 Hz). A multiplet appears between 7.14 to 7.24 ppm accounting for five hydrogens. There are seven CH carbons at 112.6, 116.8, 118.2, 119.6, 121.1, 128.5, and 129.1 ppm. Two CH carbon signals appear at 128.1 ppm (merging together). The quaternary carbons appear at 110.7, 128.9, 134.9, 136.1, 136.3, 136.5, 138.2, and 140.1 ppm.  Table 5 reveals the homonuclear and heteronuclear connectivities of different nuclei in this compound. Assignment of different carbons and hydrogens for compound (**IIa**) based on the connectivity studies is shown in Figure 4.

It is clear that the phenyl substituent at the olefinic carbon goes out of plane to the rest of the ring. This ultimately leads to the shielding of the* peri* hydrogen and also the other olefinic hydrogen, with the ring current effect being responsible for this shielding. The crystal structure also confirms this; the phenyl ring is twisted by nearly 38°. The olefinic hydrogen (5.39 ppm) and the benzylic hydrogen (3.31 ppm) have poor coupling between them indicating that they may probably be nearly orthogonal to each other. This is confirmed in the crystal structure. The considerable coupling of 12.0 Hz between the benzylic hydrogen and the ring junction hydrogen (3.06 ppm) amounts to that they are antiparallel to each other. This is again found to be corroborated in the solid state, with the torsion angle between these hydrogens being 177.6°. This ring junction hydrogen has a broad triplet with coupling nearly 11.7 Hz suggesting that it assumes an axial-like arrangement with another axial coupling with the ring vicinal hydrogen. The inspection of the crystal structure suggests this could be the axial hydrogen vicinal to the ring junction hydrogen which also experiences the anisotropic shielding of the *p*-methylphenyl ring.

**Figure 5:**
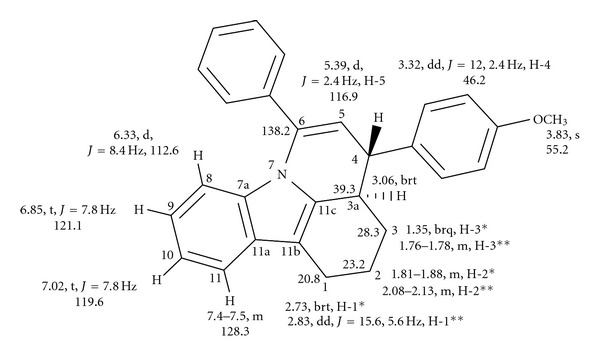
The assignment of different carbons and hydrogens of compound (**IIb**) based on the connectivity studies.

The NMR data for compound (**IIb**) are very similar to that of compound (**IIa**), and Table 6 summarizes the connectivity observed in the two-dimensional NMR spectra of (**IIb**), and Figure 5 indicates the assignment of different carbons and hydrogens of (**IIb**). The significant IR bands observed for (**IIb**) are 1428, 1450, 1504, 1567, 1619, 2922, 2835, 3016, and 3043 cm^−1^.

### 3.3. Crystal Structure

The pyrido ring of both the compounds adopts a twist boat conformation. The carbazole molecule of compounds (**IIa**) and (**IIb**) has the twisted envelope structure with atoms C3 and C13 at the flap, respectively, and they have the distance of 0.777(3) Å and 0.651(2) Å from the mean plane of the carbazole. The most favoured orientation of the phenyl and methylphenyl/methoxyphenyl rings in the structure is that their least-square mean planes are inclined toward the opposite directions with respect to least-square mean plane of the carbazole rings and they are essentially planar and perpendicular to each other by the planar angles, 80.72(3)° and 82.33(3)°, in compounds (**IIa)** and (**IIb),** respectively. In the crystal structure of (**IIa)**, H3A and H4 have the transconformation with the torsion angle of 176.60(2)°. In compound (**IIb)** too, similar conformation is found. The structures are stabilized by weak intermolecular interactions. Further, no classical hydrogen bonds are observed in the crystal structure. From the crystal structures, it is seen that there is no change in the ideal nature of the moiety due to the substitution of methylphenyl or methoxyphenyl groups. 

## Figures and Tables

**Table 1:** The crystal data, experimental conditions and structure refinement parameters for the compound **(IIa)**.

Empirical formula	C_28_H_25_N
Formula weight	375.49
Temperature	293(2) K
Wavelength	0.71073 Å
Crystal system, space group	Triclinic, P-1
Unit cell dimensions	*a* = 8.936(2) Å, *α* = 102.69(5)°
	*b* = 10.490(1) Å, *β* = 103.27(3)°
	*c* = 11.801(1) Å, *γ* = 93.80(1)°
Volume	1042.4(4) Å^3^
*Z*, Calculated density	2, 1.196 Mg/m^3^
Absorption coefficient	0.069 mm^−1^
*F*(000)	400
Crystal size	0.29 × 0.23 × 0.21 mm^3^
Theta range for data collection	2 to 25°
Limiting indices	−1 ≤ *h* ≤ 10, −12 ≤ *k* ≤ 12, −14 ≤ *l* ≤ 13
Reflections collected/unique	4444/3669 (*R*_int⁡_ = 0.0134)
Completeness to *θ* = 24.97	100.0%
Absorption correction	Psi-scan
Refinement method	Full-matrix least-squares on *F* ^2^
Data/restraints/parameters	3669/0/264
Goodness-of-fit on *F* ^2^	1.337
Final *R* indices (*I* > 2*σ*(*I*))	*R*_1_ = 0.0419, *wR*_2_ = 0.1655
*R* indices (all data)	*R*_1_ = 0.0568, *wR*_2_ = 0.1737
Extinction coefficient	0.027(4)
Largest diff. peak and hole	0.221 and −0.158 e·Å^−3^

**Table 2:** The crystal data, experimental conditions, and structure refinement parameters for the compound **(IIb)**.

Empirical formula	C_28_H_25_NO
Formula weight	391.49
Temperature	293(2) K
Wavelength	0.71069 Å
Crystal system, Space group	Monoclinic, P2_1_/a
Unit cell dimensions	*a* = 11.376(5) Å
	*b* = 14.139(5) Å, *β* = 97.413(5)°
	*c* = 13.237(5) Å
Volume	2111.3(14) Å^3^
*Z*, Calculated density	4, 1.232 Mg/m^3^
Absorption coefficient	0.074 mm^−1^
*F*(000)	832
Crystal size	0.23 × 0.19 × 0.13 mm^3^
Theta range for data collection	2 to 25°
Index ranges	0 ≤ *h* ≤ 13, −1 ≤ *k* ≤ 16, −15 ≤ *l* ≤ 15
Reflections collected	4245
Independent reflections	3685 (*R*_int⁡_ = 0.0189)
Completeness to *θ* = 24.96°	99.6%
Absorption correction	Psi-scan
Refinement method	Full-matrix least-squares on *F* ^2^
Data/restraints/parameters	3685/0/272
Goodness-of-fit on *F* ^2^	1.078
Final *R* indices [*I* > 2*σ*(*I*)]	*R*_1_ = 0.0379, *wR*_2_ = 0.0970
*R* indices (all data)	*R*_1_ = 0.0755, *wR*_2_ = 0.1214
Extinction coefficient	0.008(1)
Largest diff. peak and hole	0.208 and −0.180 e·Å^−3^

**Table 3:** Some important bond lengths (Å) and bond angles (°) for compound **(IIa)**.

N(7)-C(11C)	1.396(3)
N(7)-C(7A)	1.401(3)
N(7)-C(6)	1.420(3)
C(11C)-C(11B)	1.350(3)
C(11C)-C(3A)	1.485(3)
C(11B)-C(11A)	1.430(4)
C(11B)-C(1)	1.494(4)
C(3)-C(2)	1.524(4)
C(2)-C(1)	1.525(4)
C(11C)-N(7)-C(7A)	107.14(19)
C(11C)-N(7)-C(6)	120.06(19)
C(18)-C(4)-C(5)	113.92(19)
C(18)-C(4)-C(3A)	112.60(19)
C(5)-C(4)-C(3A)	108.36(18)
C(11B)-C(11C)-C(3A)	127.5(2)
N(7)-C(11C)-C(3A)	121.1(2)
C(17)-C(12)- C(6)	121.4(2)
C(6)-C(5)-C(4)	122.9(2)
C(5)-C(6)-N(7)	117.5(2)
C(5)-C(6)-C(12)	122.8(2)
C(19)-C(18)-C(4)	121.6(2)
C(11C)-C(3A)-C(3)	107.1(2)
C(11C)-C(3A)-C(4)	107.83(19)
C(3)-C(3A)-C(4)	114.94(19)
C(11C)-C(11B)-C(1)	122.5(2)
C(11A)-C(11B)-C(1)	130.9(2)
C(2)-C(3)-C(3A)	111.5(2)
C(3)-C(2)-C(1)	111.7(2)
C(11B)-C(1)-C(2)	110.1(2)

**Table 4:** Some important bond lengths (Å) and bond angles (°) for compound **(IIb)**.

N(1)-C(10)	1.398(2)
N(1)-C(11)	1.415(2)
C(10)-C(9)	1.484(3)
O(1)-C(2)	1.374(3)
O(1)-C(1)	1.417(3)
C(9)-C(13)	1.532(3)
C(9)-C(8)	1.543(3)
C(8)-C(12)	1.525(3)
C(16)-C(15)	1.497(3)
C(13)-C(14)	1.526(3)
C(14)-C(15)	1.531(3)
C(12)-C(11)-N(1)	117.88(17)
C(12)-C(11)-C(23)	122.99(18)
N(1)-C(11)-C(23)	118.79(17)
C(16)-C(10)-C(9)	127.77(18)
N(1)-C(10)-C(9)	121.28(16)
C(2)-O(1)-C(1)	117.8(2)
C(10)-C(9)-C(13)	107.09(15)
C(10)-C(9)-C(8)	109.10(16)
C(13)-C(9)-C(8)	114.90(16)
C(5)-C(8)-C(12)	114.82(16)
C(5)-C(8)-C(9)	111.68(16)
C(12)-C(8)-C(9)	107.74(16)
C(10)-C(16)-C(15)	122.36(19)
C(17)-C(16)-C(15)	130.74(18)
C(14)-C(13)-C(9)	111.10(17)
O(1)-C(2)-C(3)	115.6(2)
O(1)-C(2)-C(7)	125.0(2)
C(11)-C(12)-C(8)	124.04(18)
C(13)-C(14)-C(15)	111.84(18)
C(16)-C(15)-C(14)	109.72(17)

**Table 5:** Two-dimensional NMR data revealing connectivities of different nuclei in compound **(IIa)**.

Proton	Chemical shifts (ppm)	C,H-COSY connections	H,H-COSY connections	HMBC connections
H-1*	2.73 (brt)	21.1 (C-1)	1.75–1.78, 2.07–2.12	110.7
H-1**	2.82(dd, *J* = 15.6, 5.8 Hz)		1.75–1.78, 2.07–2.12	23.2, 28.3, 110.7, 136.5, 136.1
H-2*	1.80–1.87 (m)	23.2 (C-2)	1.34	21.1, 39.2, 136.5
H-2**	2.07–2.12 (m)		2.73, 2.82	21.1, 110.7
H-3*	1.34 (brq)	28.3 (C-3)	1.75–1.78, 3.04	23.2
H-3**	1.75–1.78 (m)		2.07–2.12	21.1, 39.2, 136.1,136.5
H-3a	3.06 (brt)	39.2 (C-3a)	1.34	—
H-4	3.31(dd, *J* = 12.2, 2.4 Hz)	46.6 (C-4)	3.04	28.3, 39.2, 116.8, 128.1, 128.9, 138.2
H-5	5.39(d, *J* = 2.4 Hz)	116.9 (C-5)	3.31	39.2, 46.6, 134.9, 138.2, 140.1
H-8	6.33(d, *J* = 8.1 Hz)	112.6 (C-8)	6.85	119.6, 128.9
H-9	6.85(t, *J* = 7.8 Hz)	121.1 (C-9)	6.33	116.1, 134.9
H-10	7.02(t, *J* = 7.8 Hz)	119.6 (C-10)	6.85	112.6, 128.9
H-11	7.40–7.50 (m)***	128.3	7.02	21.1, 110.7, 121.1, 128.9, 135.0

*Axial like

**Equatorial like

***Appears along with other aromatic hydrogens.

**Table 6:** Two-dimensional NMR data revealing connectivities of different nuclei in compound **(IIb)**.

Proton	Chemical Shifts (ppm)	C,H-COSY connections	H,H-COSY connections	HMBC connections
H-1*	2.73 (brt)	20.8 (C-1)	1.81–1.88, 2.08–2.13	110.7
H-1**	2.83(dd, *J* = 15.6, 5.6 Hz)		1.81–1.88, 2.08–2.13	23.2, 28.3, 110.7, 136.1, 136.5
H-2*	1.81–1.88 (m)	23.2 (C-2)	1.35	21.1, 39.2, 136.5
H-2**	2.08–2.13 (m)		2.73, 2.83	—
H-3*	1.35 (brq)	28.3 (C-3)	1.81–1.88, 3.06	23.2
H-3**	1.76–1.78 (m)		2.08–2.13	21.1, 39.2, 136.5
H-3a	3.06 (brt)	39.3 (C-3a)	1.35	46.2
H-4	3.32(dd, *J* = 12.0, 2.4 Hz)	46.2 (C-4)	3.06	28.3, 39.2, 116.9, 128.1, 128.9, 138.2
H-5	5.39(d, *J* = 2.4 Hz)	116.9 (C-5)	3.32	39.3, 46.2, 134.9, 138.2, 140.1
H-8	6.33(d, *J* = 8.4 Hz)	112.6 (C-8)	6.85	119.6, 128.9
H-9	6.85***(t, *J* = 7.8 Hz)	121.1 (C-9)	7.02	
H-10	7.02(t, *J* = 7.8 Hz)	119.6 (C-10)	7.40–7.50	112.6, 128.9
H-11	7.40–7.50 (m)***	128.3	7.02	110.7, 121.1, 128.9, 135.0

*Axial like

**Equatorial like

***Appears along with other aromatic hydrogens.
